# Popular Lectures on Cancer

**Published:** 1923-11

**Authors:** 


### POPULAR LECTURES ON CANCER.

ON the dreaded subject of cancer popular lectures till very lately have been deemed unfit for the laity even by many medical men who know the need for encouraging the public to seek advice in time when any, even benign, tumour fails to disperse naturally. Special interest therefore attaches to the Health Week just concluded at St. Pancras under the initiative of the acting Medical Officer of Health, Dr. Stella Churchill, for the week concluded with a lantern lecture on "Cancer," by Dr. Lazarus-Barlow, Professor of Experimental Pathology at the Middlesex Hospital. The effect upon the audience was no less instructive than the lecture itself.

A PERMANENT EDUCATIONAL CAMPAIGN.

Dr. Lazarus-Barlow is a practised speaker, and has very recently delivered a similar lecture at Sheffield before an audience of 2,000. He told us that half as many again had been turned away. At St. Pancras, which, unlike Sheffield, has not been nursed yet into a mood of educational zeal, the audience numbered between sixty and seventy. But this will prove the acorn to the oak if the Mayor is able to carry out his expressed desire to make this, the first Health Week held in the borough, the beginning of a permanent educational campaign with the Town Hall for its appropriate centre. He and Dr. Churchill can succeed in this admirable aim, without which Health Weeks prove abortive, if they continue to co-operate. The audience was deeply interested, and probably carried away Dr. Lazarus-Barlow's chief lesson—that hopes and fears about cancer alike yield to something more solid and useful so soon as people will learn the elementary facts and so take the elementary precautions dictated by them; these last the doctors, when consulted in time, are, of course, anxious to further.

SOFTENING THE SHOCK.

One minor lesson deserves a word. Slides of microscopic sections appear ugly and alarming to those previously unfamiliar with their appearance; an innocent plaster contour-map, with mountains in relief, has been known to have a similar effect on children's imaginations. We therefore suggest that, before these slides are shown, a moment should be spent to throw on the screen a slide depicting the density of population in a city. As we all know, such a slide looks curiously like a section of cancerous proliferation. It might prepare the audience for the visual appearance of other slides and so overcome its members' uneasy sense of strangeness. This is a detail worth considering. The lecture itself was admirably clear, informing, and reassuring in the scientific sense. We hope that Dr. Lazarus-Barlow will be invited to repeat it again and again at other centres, now that St. Pancras has given to London a useful lead.

---

**Miners' Help Coventry Hospital.**

The Warwickshire Miners' District Welfare Fund has given £1,000 toward the debt on the extension fund at the Coventry and Warwickshire General Hospital. The gift will probably be used to name a bed in commemoration of the generous donors.

